# The Formation of Metal Hydrides on the Surface of Spherical Structures and the Numerical Evaluation of the Hydrogenation Process

**DOI:** 10.3390/ma18112595

**Published:** 2025-06-02

**Authors:** Zulfiqar Khalil, Žydrūnas Kavaliauskas

**Affiliations:** Plasma Processing Laboratory, Lithuanian Energy Institute, Breslaujos Str. 3, LT-44403 Kaunas, Lithuania; zydrunas.kavaliauskas@lei.lt

**Keywords:** metal hydrides, hydrogen storage, mathematical model, Python programming language, solid-state hydrogen storage

## Abstract

Hydrogen possesses distinctive characteristics that position it as a potential energy carrier to substitute fossil fuels. Nonetheless, there is still an essential need to create secure and effective storage solutions prior to its broad application. The use of hydride-forming metals (HFMs) for hydrogen storage is a method that has been researched thoroughly over the past several decades. This study investigates the structural and chemical modifications in titanium (Ti) and zirconium (Zr) thin coatings over aluminum hydroxide (AlO_3_) granules before and after hydrogenation. The materials were subjected to hydrogenation at 400 °C and 5 atm of hydrogen pressure for 2 h, with a hydrogen flow rate of 0.8 L/min. The SEM analysis revealed significant morphological changes, including surface roughening, a grain boundary separation, and microcrack formations, indicating the formation of metal hydrides. The EDS analysis showed a reduction in Ti and Zr contents post-hydrogenation, likely due to the formation of hydrides. The presence of hydride phases, with shifts in diffraction peaks indicating structural modifications due to hydrogen absorption, is confirmed by the XRD analysis. The FTIR analysis revealed dihydroxylation, with the removal of surface hydroxyl groups and the formation of new metal–hydride bonds, further corroborating the structural changes. The formation of metal hydrides was confirmed by the emergence of new peaks within the 1100–1200 cm^−1^ range, suggesting the incorporation of hydrogen. Mathematical modeling based on the experimental parameters was conducted to assess the hydride formation and the rate of hydrogen penetration. The hydride conversion rate for Ti- and Zr-coated AlO_3_ granules was determined to be 3.5% and 1.6%, respectively. While, the hydrogen penetration depth for Ti- and Zr-coated AlO_3_ granules over a time of 2 h was found to be 1200 nm and 850 nm approximately. The findings had a good agreement with the experimental results. These results highlight the impact of hydrogenation on the microstructure and chemical composition of Ti- and Zr-coated AlO_3_, shedding light on potential applications in hydrogen storage and related fields.

## 1. Introduction

Syngas, primarily composed of hydrogen (H_2_) and carbon monoxide (CO), serves as a key feedstock for the production of various chemicals and liquid fuels through processes such as methanol synthesis and Fischer–Tropsch synthesis. It can be produced from a wide range of sources, including natural gas, coal, biomass, and virtually any hydrocarbon-based feedstock. While coal was historically the dominant raw material, modern technologies enable syngas generation from diverse inputs. This is typically achieved through reactions with steam (steam reforming), carbon dioxide (dry reforming), or oxygen (partial oxidation) [1,2,3,4,5,6].

Hydrogen possesses distinctive features that render it a possible energy vector to supplant fossil fuels. Hydrogen is a prevalent element, typically combined with other elements to produce compounds such as water and organic substances. Molecular hydrogen (H_2_) is a very light, non-toxic gas that can be utilized to power fuel cells and combustion engines as well as produce electricity under normal circumstances. Molecular hydrogen possesses an exceptionally high gravimetric energy density, approximately threefold greater than that of typical fuels like petrol (with a lower heating value of 33.33 vs. 12.40 kWh kg^−1^) [7]. Hydrogen may emerge as a significant energy vector in the coming years, particularly when integrated with renewable energy sources like solar and wind, offering a solution for the constant supply of created energy [8]. In contrast to fossil fuels, hydrogen is not naturally found in nature. It can be crafted from any primary energy source and subsequently utilized as fuel for either direct combustion in an internal combustion engine or in a fuel cell, generating just water as a byproduct [9,10,11,12]. A diverse array of techniques exists for hydrogen production, which can be categorized into two primary types based on the raw materials utilized: conventional and renewable technologies. The conventional category involves the processing of fossil fuels and encompasses hydrocarbon reforming and pyrolysis technologies. The hydrocarbon-reforming process involves the chemical processes of steam reforming, partial oxidation, and autothermal steam reforming. The second group includes techniques that generate hydrogen from renewable sources, specifically biomass or water. These technologies, employing biomass as a feedstock, can be classified into two primary subcategories, i.e., thermochemical and biological processes. The second category of renewable technologies pertains to systems that generate H_2_ by water-splitting processes, including electrolysis, thermolysis, and photo-electrolysis, employing water as the sole material input [13,14].

Currently, there are three primary methods for hydrogen storage: high-pressure gas storage, cryogenic liquid storage, and solid storage using metal hydrides. Compressing hydrogen at 15–70 MPa represents the most advanced technology. Nevertheless, it possesses a modest volumetric density (10–40 kg m^−3^) [15,16]. Liquefying hydrogen enhances its volumetric density to around 70 kg m^−3^; nonetheless, the process is energy-intensive, requiring 12.0–15.5 kWh per kilogram of H_2_. The boiling-off of hydrogen presents safety concerns. An alternate method involves utilizing metal hydrides to store hydrogen under mild circumstances, such as at or near room temperature. Numerous metal and complex hydrides have been created over the years, allowing for the selection of hydrides with specified properties based on application needs [17]. Metal hydrides consist of metal atoms containing a host lattice and hydrogen atoms. Metals with hydrogen typically generate two distinct types of hydrides: the α-phase, characterized by partial hydrogen absorption, and the β-phase, in which the hydride is completely created [18]. Metal hydrides exhibit a superior hydrogen storage density (6.5 H atoms/cm^3^ for MgH_2_) compared to hydrogen gas (0.99 H atoms/cm^3^) or liquid hydrogen (4.2 H atoms/cm^3^) [19]. Consequently, metal hydride storage provides a secure and space-efficient approach for on-board vehicle applications. Direct dissociative chemisorption and electrochemical water splitting are two approaches that could be used to hydrate a metal.

These reactions are as follows:

(1) $$M+\frac{x}{2}H_{2}\;↔\;MH_{x}\;$$

(2) $$M+\frac{x}{2}H_{2}O+\frac{x}{2}e^{-}\;↔\;MH_{x}+\frac{x}{2}{OH}^{-}$$

where M represents the metal.

Hydrogenation begins with the dissociative chemisorption of surface H_2_ molecules and continues with the diffusion of bulk H atoms into the metal matrix through interstitials, setting the stage for the production of hydrides. The outcome is the creation of a solid solution (α-phase) of hydrogen within the host metal, which occurs between the interstitial spaces. A phase transition occurs in the M-H system when the concentration of hydrogen in the α-solid solution rises, leading to the formation of the hydride (β-phase) with a high H/M ratio and a hydrogen sublattice that is either partially ordered (binary hydrides) or completely ordered (intermetallic hydrides) [20].

Flat surface hydrides are essential in applications necessitating stability, uniformity, and regulated hydrogen interactions. Although, they may be deficient in large storage capacities and rapid kinetics compared to nanostructured materials. The production of metal hydride on a flat surface entails processes such as adsorption, diffusion, and nucleation and growth. The surface’s flatness can affect the kinetics of these stages. Research indicates that subsurface hydride production can result in diminished surface adsorption kinetics, hence impacting the overall efficiency of the process [21]. Conversely, the spherical morphology of the granules can affect these stages by offering a consistent surface area and reducing stress concentrations during hydride production. For the advancement of hydrogen storage technologies, this approach is a worthwhile research topic due to its benefits in kinetics, packing density, structural integrity, and thermal management [22,23].

In our study, Zr (zirconium) and Ti (titanium) and their coating over AlO_3_ (aluminum hydroxide) granules was chosen for hydride formation using magnetron sputtering technology. The uniqueness of this work is twofold: (1) The dynamics of hydride formation on the surface of spherical granules were studied, rather than on flat substrates, as in many previous studies and (2) this study combines experimental characterization and mathematical modeling to evaluate hydride’s real diffusion and degree under specific conditions (400 °C, 5 atm H_2_). This combination, especially when using spherical granules, allows us to evaluate the possibilities of practical applications in hydrogen storage systems, where materials of this shape are often used due to their better surface area and heat exchange. AlO_3_ granules act as a spatial structural framework (3D skeleton structure), allowing hydrogen gas to flow freely through the gaps between the granules while ensuring a sufficient contact between the gas and the active (Ti or Zr) metal coating deposited on the AlO_3_ surface. In this way, local conditions were created for the formation of hydrides in those places where hydrogen reaches the metal through the spatial structure formed by AlO_3_ granules with a spherical surface. Zirconium (Zr) and titanium (Ti) were selected due to their superior hydrogen absorption characteristics and their ability to form thermodynamically stable hydride phases, specifically TiH_2_ and ZrH_2_. Both metals demonstrate a high hydrogen absorption efficiency, low dehydration temperatures, a strong durability, and excellent hydrogen penetration capabilities. Additionally, Zr and Ti exhibit corrosion resistance and form stable metal hydride structures, making them reliable and cost-effective. Their favorable activation energy profiles and widespread applications in nuclear and energy storage industries further emphasize their relevance to contemporary engineering challenges, while magnetron sputtering is used to form coatings as it allows the creation of thin, uniform coatings with better structural properties.

This study aims to form Zr (zirconium) and Ti (titanium) hydrides on spherical aluminum hydroxide granules and investigate their hydrogen absorption and structure formation properties along with conducting mathematical modeling based on the experimental parameters to assess hydride formation and the rate of the hydrogen penetration. The current literature reveals a significant lack of information on the influence of spherical substrates on hydride formation, as there is almost no experimental and mathematical modeling data to study this phenomenon. This study advances the understanding of how microstructural features (microcracks and grain boundaries) impact hydride development. Furthermore, a diffusion model is presented, enabling the prediction of reaction rates and penetration depths, which are critical for the design of efficient hydrogen storage devices. In addition, we have shown that the 3D granule-form structure can be applied in hydrogen storage systems.

## 2. Materials and Methods

**Figure 1:**
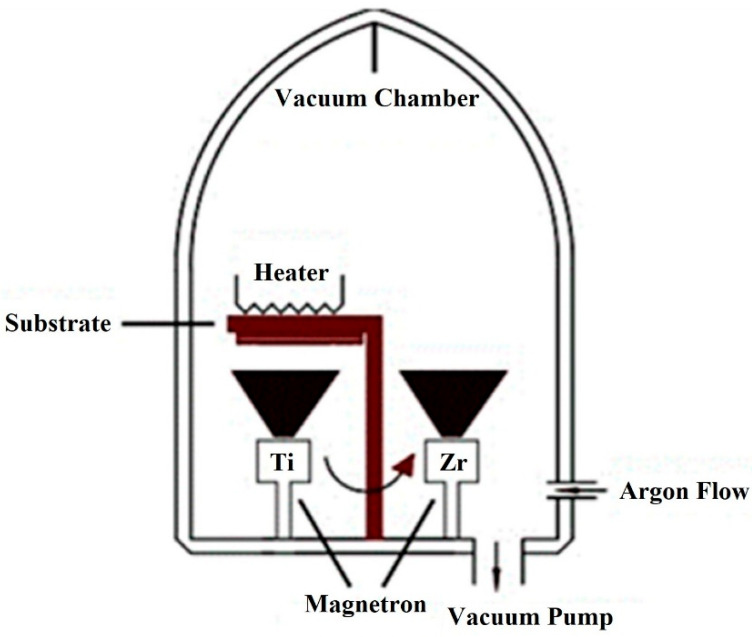
Schematic representation of magnetron sputtering equipment.

Magnetron sputtering technology was used to form the Zr and Ti coatings over AlO_3_ granules. Zr and Ti layers’ structures were grown utilizing DC magnetron sputtering within an argon gas environment, maintaining a pressure (p) of 1.33 Pa to ensure deposition process stability. To regulate the gas environment, an argon flow meter connected to the chamber enabled the adjustment of argon gas flow rates within the range of 500 cm^3^/min. The Zr and Ti disk targets, each possessing a 3-inch diameter, were sourced from Kurt J. Lesker Company in Jefferson Hills, PA, USA, with a noteworthy purity of 99.95%. The spatial arrangement of the deposition system involved maintaining a constant distance of 6 cm between the magnetrons and the substrate, as illustrated in Figure 1. Notably, the fixed target currents for Zr and Ti were set at 0.5 A, respectively. During the magnetron deposition process, the samples were subjected to a controlled heating mechanism, maintaining a substrate temperature of 423 K. Elevating the substrate temperature serves a dual purpose: enhancing adhesion between the deposited layers and the substrate and augmenting the energy and mobility of atoms on the substrate surface. This strategic manipulation of temperature contributes to the overall quality and characteristics of the deposited layer structures. The deposition times for Zr and Ti were precisely delineated at 10 min, respectively. This temporal control plays a pivotal role in determining the properties of the resultant layers, ensuring a tailored approach to achieving the desired layer structure. To ensure uniform coating over spherical granules, distance between the source and granules was ensured. Also, granules in the chamber were constantly stirred by vibration so that each was irradiated evenly. However, some areas of the granules may be unevenly covered. In summary, this detailed deposition process involves a comprehensive control strategy, encompassing gas flow rates, pressure, target currents, substrate temperature, and deposition times. For the hydrogenation process, granules of aluminum hydroxide coated with zirconium (Zr) and titanium (Ti) were placed in a reaction chamber, separated by cotton wool to prevent direct contact between them. The reaction chamber was connected to a hydrogen gas source at one end and sealed at the other. It was then placed inside an Elite thermal system equipped with three heaters positioned on the right, left, and center to ensure uniform temperature distribution throughout the chamber. All heaters were set to a temperature of 400 °C. Hydrogen gas was introduced at a constant flow rate of 0.8 L/min under a pressure of 5 atm for a duration of 2 h. These parameters were selected based on literature reports to achieve optimal hydrogenation conditions.

A Hitachi S-3400 N model scanning electron microscope (SEM) was used to evaluate the morphology and geometric characteristics of the primary material and the obtained product after the hydrogenation. The actual thickness of Zr and Ti coatings is challenging to determine since these layers were formed on round-structured AlO_3_ granules. Therefore, it is almost impossible to produce cross-section SEM images, which can be easily obtained on a flat surface, to estimate the coating thickness. The approximate thickness of the coating can reach up to 1000 nm or more. The energy-dispersive X-ray spectroscopy (EDS) method was used to evaluate the elemental composition of the raw material and post-processing products and was performed in conjunction with surface scanning electron microscopy. X-ray diffraction (XRD) with standard Bragg–Brentano focusing geometry was used to study the raw material and the post-processed material (to determine the crystalline structure and predominant compounds). Fourier transform infrared spectroscopy (FTIR) was used to evaluate the functional groups in the raw material (Zr and Ti aluminum hydroxide) and the material after processing. By analyzing the FTIR spectra, the changes in the characteristic peaks of organic compounds were evaluated, revealing the dynamics of substances of organic origin that appear during their formation.

An advanced Python 3.9 program code was created to derive conclusions based on advanced kinetic and diffusion equations. The algorithm utilized Arrhenius-type equations to compute the reaction rate constant, effective rate correction based on coating thickness, and an advanced kinetic model to assess the degree of hydride production over time. The extent of hydrogen penetration was determined using the classical diffusion equation, which is dependent upon time and the diffusion coefficient. The values of the diffusion and kinetic parameters used for the simulation were selected based on literature data, corresponding to the formation properties of titanium (Ti) and zirconium (Zr) metal hydrides. The diffusion coefficient (D), the activation energy (Ea), and the pre-exponential Arrhenius factor (A) were selected based on data from Wipf et al. 2000 [24], which present experimental values for hydrogen diffusion through Ti and Zr and are as follows:

$$\begin{array}{cc} \left.\begin{array}{l} D=1\times 10^{-16}\;m^{2}/s\\ A=1\times 10^{3}\;\min^{-1}\\ \mathrm{Ea}=85\;\mathrm{kJ}/\mathrm{mol} \end{array}\right\} & \mathrm{For}\;\mathrm{Ti}\\ \left.\begin{array}{l} D=5\times 10^{-17}\;m^{2}/s\\ A=8\times 10^{2}\;\min^{-1}\\ \mathrm{Ea}=95\;\mathrm{kJ}/\mathrm{mol} \end{array}\right\} & \mathrm{For}\;\mathrm{Zr} \end{array}$$

These values were introduced into an advanced kinetic model that allows the calculation of the hydride formation rate and includes the classical diffusion equation ($x=\sqrt{2Dt}$), which was used to estimate the depth of hydrogen penetration.

The formulas were utilized for both titanium and zirconium coatings, and the findings were illustrated in graphs that distinctly demonstrated the disparities between these two materials regarding hydrogen penetration rates and hydride creation. There is one main assumption, i.e., that 1D hydrogen diffusion is used in the modeling. Diffusion is assumed to occur solely through the depth of a thin, homogeneous coating perpendicular to the surface. This simplification was selected for the following reasons:

- The titanium and zirconium coatings analyzed in this study are extremely thin, so the diffusion occurred mainly in the depth direction and not along the surface, this justifies the use of a 1D diffusion model.
- The objective of the model was to enable a direct comparison between the diffusion behaviors of Ti and Zr under identical conditions. To ensure consistency and minimize complexity, a simplified 1D framework was adopted.
- The diffusion coefficient was considered constant throughout the diffusion process, independent of depth and concentration, hence, allowing us to use the classical diffusion equation ($x=\sqrt{2Dt}$).

Although the real system has a 3D spherical bead geometry, the 1D model was deemed a suitable first approximation for thin coatings. This approach enabled a more precise analysis by avoiding additional uncertainties related to 3D geometry and variable parameters. This mathematical modeling enabled both a quantitative assessment of the processes and a justification of the reported experimental discrepancies through theoretical calculations.

## 3. Results and Discussion

**Figure 2:**
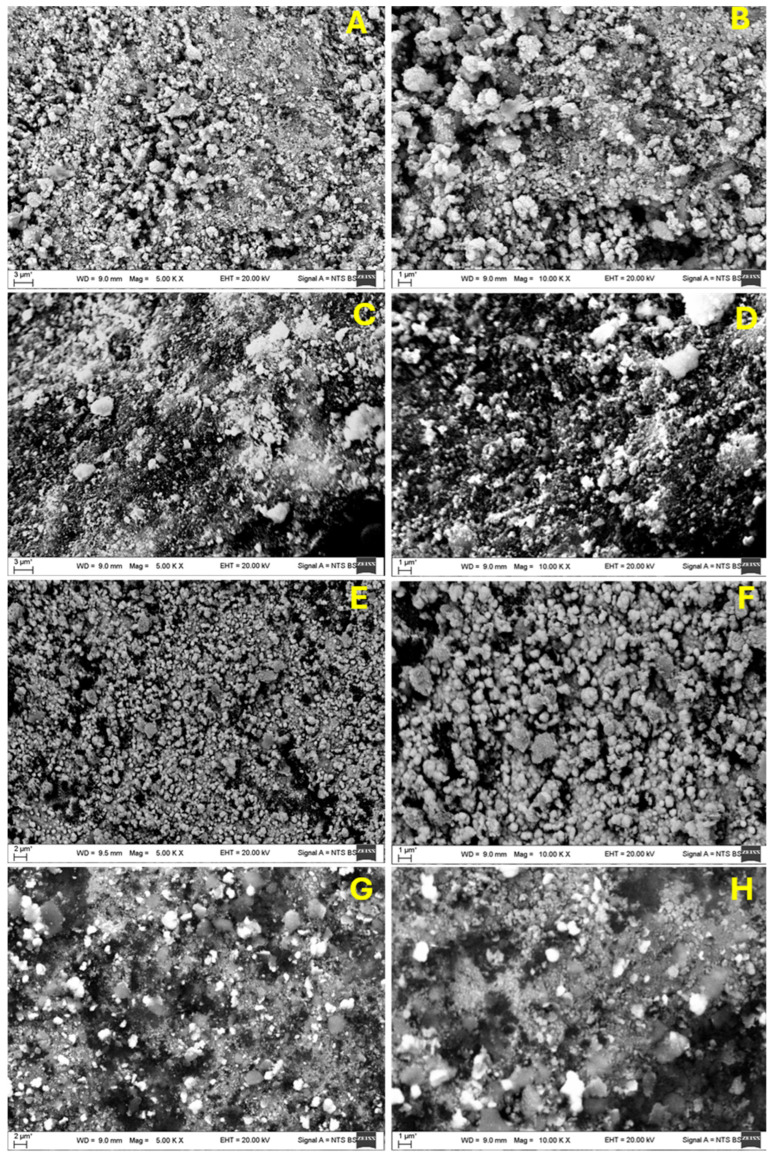
SEM images with 5 K and 10 K magnification: (**A**,**B**) Ti coating over AlO_3_ before hydrogenation, (**C**,**D**) Ti coating over AlO_3_ after hydrogenation, (**E**,**F**) Zr coating over AlO_3_ before hydrogenation, and (**G**,**H**) Zr coating over AlO_3_ after hydrogenation.

The SEM imaging of the Ti and Zr thin coatings over AlO_3_ granules before and after hydrogenation revealed significant surface morphological changes. Both materials had a stable and compact structure prior to hydrogenation, as depicted in Figure 2A,B,E,F [25], with smooth, densely packed surfaces; fine, spherical grains; and minimum porosity. Hydrogenation caused both materials to become rougher, as seen in Figure 2C,D,G,H, with a grain boundary separation and microcrack formations being particularly visible. The distribution of Ti derivatives is not uniform; rather, they are distributed unevenly, showing a clear preference for locations at intermetallic phases and grain boundaries. Furthermore, the brighter areas seen in the SEM images suggest the presence of these hydride phases or surface oxides. These phases generally possess higher atomic numbers, which can result in a brighter appearance in SEM imaging due to variations in electron scattering. The non-uniform distribution of hydrides and their tendency to form preferentially at grain boundaries indicate that the hydrogen absorption is not homogeneous and that hydrogen may move towards areas with a higher atomic density or structural imperfections [26,27]. The density numbers of the aforementioned hydride precipitates might vary for different grain orientations and are not homogeneous. The observed changes in the precipitation densities of the hydride spots within different grain orientations could be because of the different properties of the coating oxidation layers (for example, their thickness, surface properties, etc.) or because of the different stress fields that form when the hydrides start to form and grow. In addition, variances in the dislocation densities of various metallic grains could be another factor contributing to these disparities. The fact that different grains may possess different dislocation densities can also be accounted for by the observed variations in the hydrides’ clustering densities in the different grains. The hydrogen absorption and subsequent volume expansion, which causes lattice distortion and brittleness, are in agreement with these changes.

The Zr coating on the surface of the AlO_3_ sample before hydrogenation was also uniform and dense. The surface grew rougher after hydrogenation, with bigger particles dispersed randomly and bright patches indicating the production of zirconium hydride (ZrH_x_) or oxide phases. Void and microcrack formations indicated that the lattice had grown as a result of hydrogen diffusion. These changes are likely to happen because hydrogenation creates metal hydrides, which cause the volume to grow, the surface to become rough, and the structural distortion [28,29].

EDS studies facilitate the determination of the elemental composition and the distribution of individual elements over the entire area. EDS studies complement other methods (e.g., XRD, as in the case of this study), as it is possible to identify the composition more precisely. The elemental compositions before and after the hydrogenation of Zr, Ti, and aluminum hydroxide granules are presented in Table 1.

The EDS analysis of the Zr and Ti thin coatings formed on the surface of AlO_3_ granules conducted before and after hydrogenation shows considerable variations in their elemental compositions. Before the hydrogenation of the Ti coatings, the oxygen content was recorded as the highest at 42.56 wt%, with significant amounts of aluminum and carbon. Following hydrogenation, the oxygen content rose to 47.71 wt%, whereas the titanium level fell sharply from 10.27 wt% to 2.07 wt%, indicating a reduction or alteration of titanium oxide, which may have led to the formation of titanium hydrides (TiHₓ). Additionally, the carbon content dropped from 12.13 wt% to 6.08 wt%, suggesting the elimination of surface contaminants. These alterations imply that hydrogenation modified the titanium levels and decreased impurities, while the oxide structure largely remained unchanged.

However, before the hydrogenation of the Zr thin coating samples, the zirconium proportion was prominent at 39.21 wt%, accompanied by notable amounts of oxygen and aluminum. After hydrogenation, the zirconium level fell to 9.45 wt%, likely due to the production of zirconium hydrides (ZrH_2_), while the oxygen content decreased from 38.27 wt% to 35.19 wt%, suggesting the emergence of oxygen vacancies. The aluminum content rose from 18.64 wt% to 31.23 wt%, implying a potential rearrangement of the alumina structure during hydrogenation. Additionally, the carbon content diminished from 7.34 wt% to 3.83 wt%, indicating the elimination of carbon impurities.

It is also worth noting that the hydride formation alone cannot explain such a significant decrease in the Zr and Ti content after hydrogenation. Several additional factors are likely:

- The bouncing or branching of metal layers due to the volumetric expansion during hydrogenation (especially for thin layers) can cause a partial coating loss from the surface.
- The EDS analysis evaluates the surface composition, and after hydrogenation, Ti and Zr can be displaced to deeper layers due to the hydride formation and microscopic cracks, thus reducing their detectable amount at the surface.

These findings reveal that hydrogenation led to structural changes, including a reduction in zirconium and the formation of oxygen vacancies alongside a reorganization of the aluminum hydroxide matrix.

**Figure 3:**
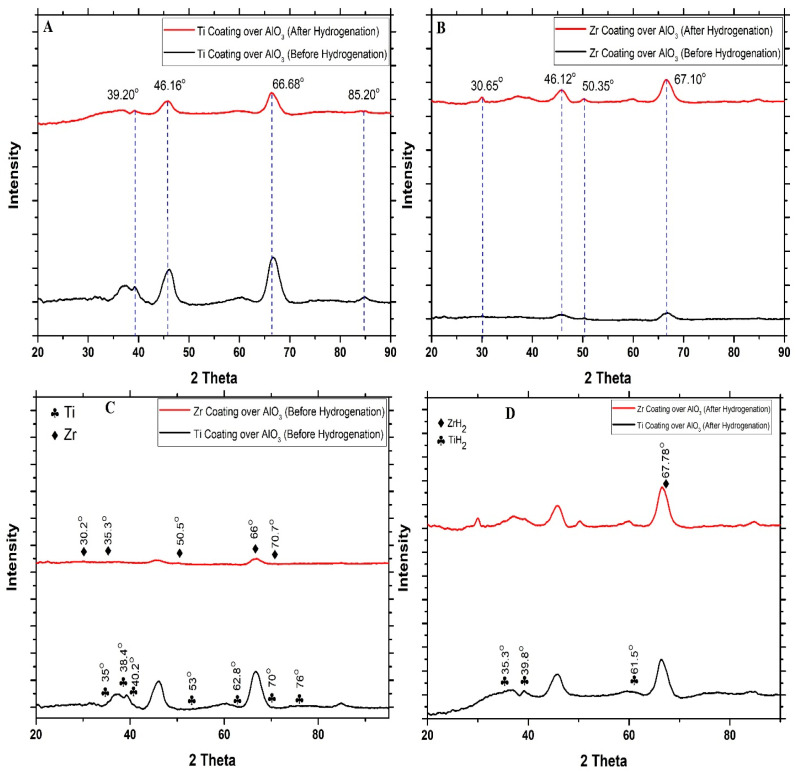
XRD graph: (**A**) Ti coating over AlO_3_ before and after hydrogenation, (**B**) Zr coating over AlO_3_ before and after hydrogenation, (**C**) Ti and Zr coating over AlO_3_ before hydrogenation, and (**D**) Ti and Zr coating over AlO_3_ after hydrogenation.

In order to examine the phase transformations of the Zr and Ti thin coating formed over the surface of the AlO_3_ granules before and after hydrogenation, an X-ray diffraction (XRD) analysis was performed (Figure 3A–D). From Figure 3A,B, it can be observed that after the hydrogenation, the peaks have moved towards a lower angle. This shift may be attributed to the incorporation of hydrogen atoms into the crystal structure, which enlarges the space between the atoms. According to Bragg’s law, this results in the diffraction peaks shifting to lower angles. The XRD spectrum before hydrogenation for both Zr- and Ti-coated samples shows either low intensity peaks or broad peaks, as shown in Figure 3C, suggesting both materials to be amorphous or poorly crystalline in their initial states. The spectrum of the Zr-coated sample is predominantly flat, suggesting that the surface is primarily non-crystalline. Peaks corresponding to Zr are approximately at 30.2°, 35.3°, 50.5°, 60.2°, 66°, and 70.7°. On the other hand, the spectrum for the Ti-coated sample displays subtle peaks, indicating the presence of minor crystalline phases. Peaks corresponding to Ti are approximately at 35°, 38.4°, 40.2°, 53°, 62.8°, 70°, and 76°.

In Figure 3D, after hydrogenation, both samples display new and more defined peaks. For the Zr coating over granules, a prominent peak appears at 2θ ≈ 67.78°, which corresponds to ZrH_2_ formation, signifying hydrogen absorption and the creation of hydrides [30,31,32,33]. Likewise, for the Ti-coated sample, peaks aligning with the typical reflections of TiH_2_ were observed to be at 35.3°, 39.8°, and 61.5° [34,35,36]. This supports the conclusion that titanium hydride is formed as a result of hydrogenation. The augmented intensity of these peaks suggests the development of crystalline hydride phases [37]. Results show that hydrogenation under high-temperature and pressure conditions facilitates the formation of metal hydrides in both materials. The XRD findings align with SEM observations, where a surface roughening and potential embrittlement were noted due to the hydride formation.

**Figure 4:**
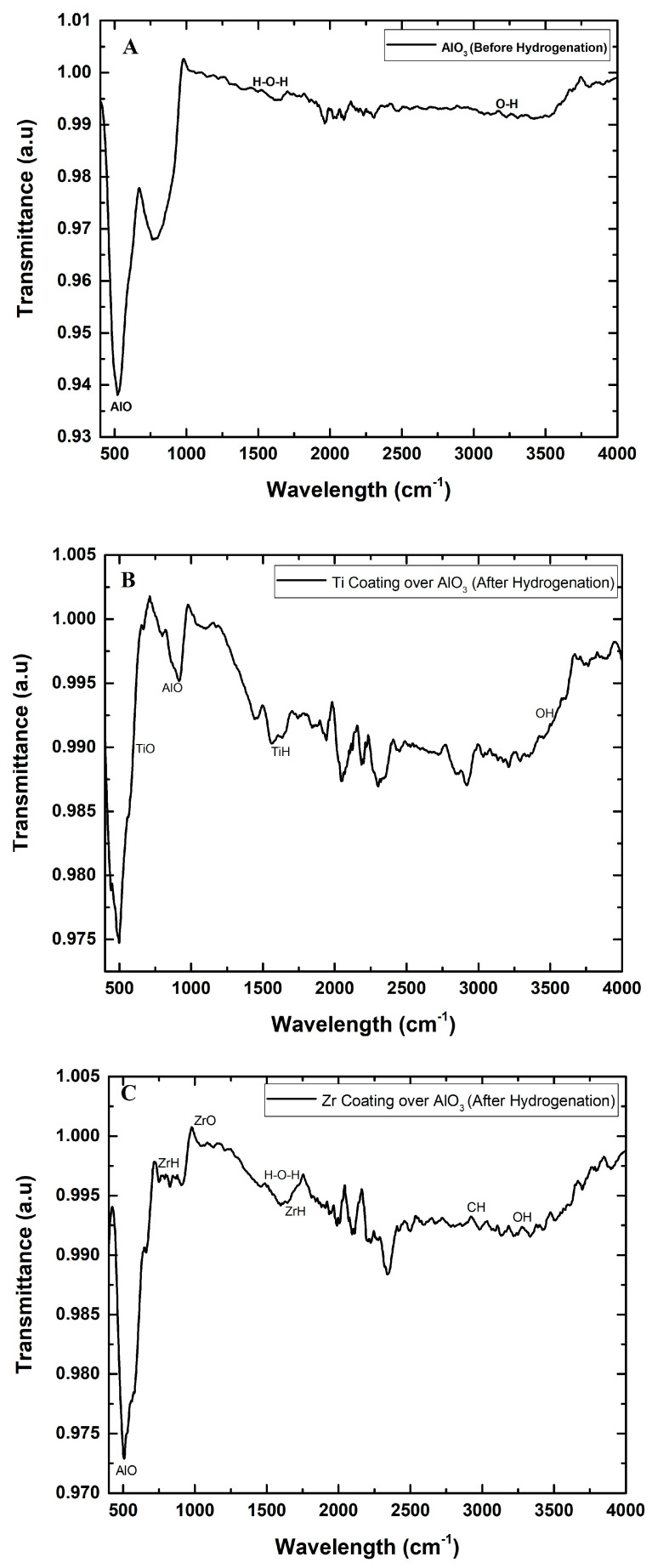
FTIR graph: (**A**) pure AlO_3_ before hydrogenation, (**B**) Ti coating over AlO_3_ after hydrogenation, and (**C**) Zr coating over AlO_3_ after hydrogenation.

The FTIR analysis of aluminum hydroxide granules before hydrogenation and Ti- and Zr-coated AlO_3_ granules after hydrogenation shows notable changes in the vibrational bands, reflecting structural and chemical modifications. Before hydrogenation, as shown in Figure 4A, AlO_3_ exhibits broad O–H stretching bands around 3000–3700 cm^−1^, indicating the presence of surface hydroxyl groups and adsorbed water. The bending vibration of H–O–H around 1630 cm^−1^ was also observed, confirming adsorbed water molecules. After hydrogenation for both Ti- and Zr-coated AlO_3_ granules, as shown in Figure 4B,C, the O–H stretching bands weakened, suggesting the dehydroxylation of the materials, likely due to the reaction with hydrogen. Attributed to Ti–O and Al–O vibrations, titanium–oxygen bonds generally show up in the range of 550–750 cm^−1^. The presence of sharp, well-defined peaks indicates the existence of crystalline Ti–O–Al structures, likely arising from solid-state interactions or the reorganization due to hydrogen exposure. Similarly, for the Zr-coated AlO_3_ granules after hydrogenation, enhanced and potentially shifted bands imply an overlap of Zr–O and Al–O bonds, suggesting a reaction between Zr and Al in the oxidized matrix. While Zr–O bands typically appear around 600–700 cm^−1^. Additionally, new peaks in the 1100–1200 cm^−1^ range can be related to the formation of ZrH (zirconium hydride) and TiH (titanium hydride). The bending and stretching vibrations of metal-hydrides (M–H, where M = Ti or Zr) often occur in the mid-IR region, but depending on the environment (e.g., oxide matrix) they can shift to the 1000–1300 cm^−1^ region. During the formation of hydrides, intermediate compounds or defects in the crystal structure can form, which change the vibrational modes and sometimes appear in a typical wavenumber region. In summary, the 1100–1200 cm^−1^ region of the FTIR spectra reflects not only the vibrations of pure hydrides, but also complex compounds that appear during the hydration process, which allows for the identification of hydride formation by an indirect, but reliable, spectroscopic method [38,39,40,41].

For both Ti and Zr and their coating over AlO_3_, shifts in the Al–O stretching bands were observed, signifying modifications in the oxide network, potentially caused by oxygen vacancies or the restructuring of the aluminum hydroxide lattice. These changes suggest that hydrogenation led to alterations in the metal–oxide bonding, with a possible hydride formation and the removal of surface hydroxyl groups. Overall, the FTIR analysis confirms that hydrogenation induces structural modifications in both Ti and Zr thin coatings formed on the surface of AlO_3_ granules, including dehydroxylation, the formation of oxygen vacancies, and changes in the metal–oxide bonding, making these materials more suited for hydrogen-related applications [42].

**Figure 5:**
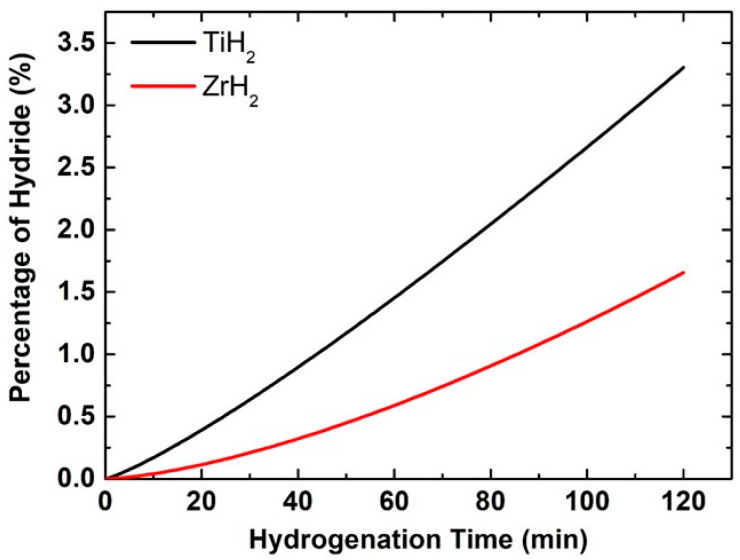
Hydride formation percentage over time.

At 400 °C and a hydrogen pressure of 5 atm, a notable disparity in hydride conversion rates is seen between titanium and zirconium coatings (refer to Figure 5). Within 2 h, titanium attains a conversion rate of roughly 3.5% to titanium hydride, but zirconium achieves just about 1.6%. This significant disparity is influenced by critical aspects pertaining to the diffusion, thermodynamic, and kinetic properties of these materials, underscoring titanium’s performance for advanced applications. At first, it is crucial to examine hydrogen diffusion, since it is a primary factor influencing the rate at which hydrogen atoms can infiltrate the metallic layer and attain the necessary depth for hydride phase formation. The hydrogen diffusion coefficient in titanium exceeds as compared to zirconium, indicating that hydrogen atoms in the titanium coating migrate more rapidly and penetrate the inner parts of the layer more swiftly [24]. Resultantly, the hydride phase formation starts earlier and disseminates more uniformly across the coating’s thickness. For zirconium, hydrogen diffusion proceeds at a reduced pace, hence constraining the reaction rate from the start. Therefore, hydrogen accumulates solely on the surface, with a limited penetration into deeper layers. Another important factor is the reaction’s activation energy, which is the minimum amount of energy needed to initiate the creation of hydride. The activation energy necessary for the reaction of titanium is approximately 85 kJ/mol, but for zirconium it is around 95 kJ/mol. This means titanium atoms react with hydrogen more rapidly due to their lower energy requirement for bond formation. This directly influences the reaction kinetics and expedites the growth of the hydride. To check the favorability of the hydride formation reaction itself, the thermodynamic aspect must also be considered. While both titanium and zirconium provide stable or metastable hydride molecules (TiH_2_ and ZrH_2_), the free energy of the reaction is marginally more negative for titanium, resulting in a more rapid and vigorous formation of the hydride phase at equivalent temperatures. Moreover, the production temperature of titanium hydrides is relatively lower than that of zirconium, making the 400 °C range more conducive to ideal circumstances for titanium. Titanium coatings may exhibit a higher prevalence of defects, grain boundaries, or pores, creating supplementary pathways for hydrogen migration inside the metal’s microstructure. This leads to an accelerated hydrogen diffusion through the layer, concurrently facilitating the reaction at various locations [43]. Conversely, zirconium may possess a denser or less advantageous structure for such migration, further reducing its reaction rate. The accelerated transformation of titanium coatings into hydrides results from enhanced diffusion characteristics, reduced activation energy, advantageous thermodynamics, and an optimal microstructure that facilitates a more rapid and efficient reaction between hydrogen and the metal. Zirconium has an inferior efficiency in all these dimensions, resulting in slower coating responses and a reduced simultaneous hydride production.

**Figure 6:**
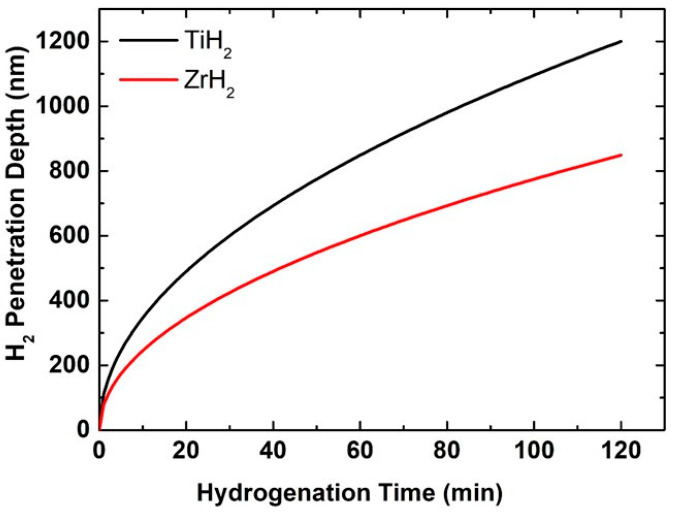
The penetration depth of hydrogen molecules into the Ti and Zr surface over time.

Under identical conditions, the penetration depth of hydrogen molecules into the titanium coating surface after 120 min reaches around 1200 nm, but into the zirconium coating surface it attains only about 850 nm (Figure 6). This difference is mainly related to the variance in hydrogen diffusion coefficients between the two metals. As a result, hydrogen atoms move faster and can more easily reach deeper layers of the coating in the titanium structure. Diffusion in metals is intricately linked to the crystal lattice type, atomic density, and the number of defects or voids present, all of which influence the mobility of diffusing particles [44]. The hexagonal close-packed (HCP) crystal structure of titanium facilitates increased gaps or diffusion pathways, allowing hydrogen migration even at moderate temperatures. Moreover, titanium often has more structural defects, such as dislocations, grain boundaries, or microscopic pores, which act as diffusion accelerators. On the other hand, zirconium, despite having an HCP structure, has a lower hydrogen diffusion coefficient. That might be because of the fact that it has small atomic spacing which is less favorable for the easy movement of the interstitial atoms. Also, the inherent oxide layer on its surface is often denser, more uniform, and more resistant to diffusion. Such surface layers can act as an additional barrier, slowing the penetration of hydrogen into the base metal. Meanwhile, the oxide layer of titanium can be thinner, more permeable, or contain microscopic defects through which hydrogen can penetrate more efficiently. It is also important to note that even at the same temperature, titanium’s lower activation energy for hydrogen diffusion means that the effect of temperature on this process will be more substantial. For example, diffusion accelerates faster than in the case of zirconium. In both cases, the thermal energy the atoms receive at a temperature of 400 °C is sufficient for the diffusion process. However, due to the aforementioned kinetic and structural factors, this energy is used more efficiently for hydrogen migration in the case of titanium. Finally, it should be noted that parameters such as the film thickness, surface roughness, or grain size also affect the penetration depth. If the titanium coating has a finer structure or more porosity, this further increases the possibilities of hydrogen penetration. For all these reasons, titanium coatings allow hydrogen to penetrate deeper.

The direct comparison of simulation results and experimental data in this case is limited due to experimental difficulties in accurately assessing the depth of the hydrogen penetration and the dynamics of the hydride formation in samples with complex structures. The samples used are spherical AlO_3_ granules coated with thin Ti and Zr coatings, which, after hydrogenation, become heterogeneous, with microholes, grain boundaries, and the local distribution of hydrides. Such topologically complex structures do not allow easy cross-sectioning for the SEM or TEM analysis because the spherical shape of the particles makes it difficult to obtain a stable section. Also, conventional preparation technologies, like FIB or ion cutting, cannot provide a representative section area for the entire particle structure. For these reasons, the theoretical model was used as an approximate but quantitatively based tool to assess general trends between different materials (Ti vs. Zr) and to understand how the course of hydration changes depending on the time, diffusion coefficient, or activation energy.

## 4. Conclusions

The hydrogenation process of Ti and Zr thin coatings over AlO_3_ granules results in significant alterations in their surface morphology and chemical makeup. The SEM analysis illustrated a notable surface roughness, grain boundary separation, and the development of microcracks, which are typical signs of metal hydride formation, while the EDS analysis indicated a decrease in the amounts of titanium and zirconium from 10.27 wt% to 2.07 wt% and from 39.21 wt% to 9.45 wt%, respectively, due to the creation of TiH_2_ and ZrH_2_ phases. The XRD analysis corroborated these results, displaying the formation of hydride phases and notable shifts in diffraction peaks, signaling lattice expansion. For the Zr coating over AlO_3_ granules, a prominent peak corresponds to the hydride and appears at 2θ ≈ 67.8°, while for the Ti coating over AlO_3_ hydrides, peaks appear at 35.3°, 39.8°, and 61.5°. The loss of surface hydroxyl groups and the establishment of metal–hydride bonds is revealed by the FTIR results, affirming the interaction between hydrogen and the surfaces of the materials. The emergence of new peaks within the 1100–1200 cm^−1^ range may be attributed to the formation of metal hydrides or alterations in the oxide matrix, suggesting the incorporation of hydrogen. Additionally, FTIR findings validate the creation of oxygen vacancies and modifications in the metal–oxide bonding, enhancing the suitability of these materials for applications related to hydrogen. The Python simulation program further confirmed these findings by accurately modeling the reaction kinetics and diffusion behavior, supporting experimental results. The hydride conversion rate for the Ti thin coating over AlO_3_ was determined to be 3.5% in 2 h, while for Zr it was approximately 1.6%. Furthermore, the hydrogen penetration depth for Ti- and Zr-coated AlO_3_ granules was found to be 1200 nm and 850 nm, respectively. The findings highlight titanium’s superior behavior in hydrogen interaction processes, as it provides a faster hydride formation and a deeper hydrogen penetration. These insights not only deepen our understanding of metal–hydrogen interactions but also provide valuable guidance for material selection in hydrogen storage and related applications.

## Figures and Tables

**Table 1:** Results of EDS tests before and after hydrogenation of Ti and Zr coating over aluminum hydroxide.

Element	Ti Coating over AlO_3_ Granules		Zr Coating over AlO_3_ Granules	
	Before Hydrogenation wt%	After Hydrogenation wt%	Before Hydrogenation wt%	After Hydrogenation wt%
Ti	10.27	2.07	0	0
Zr	0	0	39.21	9.45
O	42.56	47.71	38.27	35.19
Al	27.54	41.55	18.64	31.23
C	12.13	6.08	7.34	3.83

## Data Availability

The original contributions presented in this study are included in the article. Further inquiries can be directed to the corresponding author.
